# Age-stage, two-sex life table of *Parapoynx crisonalis* (Lepidoptera: Pyralidae) at different temperatures

**DOI:** 10.1371/journal.pone.0173380

**Published:** 2017-03-06

**Authors:** Qi Chen, Ni Li, Xing Wang, Li Ma, Jian-Bin Huang, Guo-Hua Huang

**Affiliations:** 1 Hunan Provincial Key Laboratory for Biology and Control of Plant Diseases and Insect Pests, Changsha, China; 2 College of Plant Protection, Hunan Agricultural University, Changsha, China; USDA Agricultural Research Service, UNITED STATES

## Abstract

*Parapoynx crisonalis* is an important pest of many aquatic vegetables including water chestnuts. Understanding the relationship between temperature variations and the population growth rates of *P*. *crisonalis* is essential to predicting its population dynamics in water chestnuts ponds. These relationships were examined in this study based on the age-stage, two-sex life table of *P*. *crisonalis* developed in the laboratory at 21, 24, 27, 30, 33 and 36°C. The results showed that the values of *S*_*xj*_ (age-stage–specific survival rate), *f*_*xj*_ (age-stage-specific fecundity), *l*_*x*_ (age specific survival rate) and *m*_*x*_ (age-specific fecundity) increased as the temperature rose from 21 to 27°C, then decreased from 30 to 36°C. Temperature also had a significant effect on the net reproductive rate (*R*_0_), gross reproductive rate (*GRR*), intrinsic rate of increase (*r)* and finite rate of increase (*λ*). The value of these parameters were at low levels at 21, 33, and 36°C. Further, the *r* value decreased as the temperature rose from 24 to 30°C, while the *GRR* reached its highest level at 27°C. The results indicated that optimal growth and development of *P*. *crisonalis* occurred at temperatures between 24°C to 30°C when compared to the lowest temperature (21°C) and higher temperatures of 33°C and 36°C.

## Introduction

In recent years, *Parapoynx crisonalis* (Walker) (Lepidoptera: Pyralidae) has become a persistant pest in water chestnut, *Nymphoides* and other aquatic crops, causing a considerable economic impact in China [1]. The damage caused by *P*. *crisonalis* is a result of the larval stage feeding on the leaves of cultivated aquatic plants including water chestnut, water lily and *Nymphoides*. The body of the larvae is somewhat translucent, making early detection on the aquatic plants difficult. The subsequent damage to the leaf surfaces eventually leads to substantial reductions in the plant’s ability to photosynthesize with corresponding reductions in output [2]. The species is distributed primarily in tropical/subtropical regions from Sri Lanka, India, Burma, Thailand, Indonesia, southern Japan and Australia, and has recently become established in Great Britain [3–4]. In China, *Parapoynx crisonalis* is mainly distributed in the southern regions and is especially prevalent in Jiangsu, Zhejiang, Fujian, Taiwan, Hubei, Hunan, Guangdong and Guangxi [5–7]. Its population increase appears to be influenced primarily by environmental temperatures [8].

Life tables have been identified as powerful and necessary tools for analyzing and understanding external factors such as the effect that various temperatures have on the growth, survival, reproduction, and intrinsic rate of insect populations [9]. Deevey (1947) [10], Birch (1948) [11], Southwood (1978) [12], and Carey (1993) [13] have reported on a number of different methods of analyzing life tables, many of which have been widely adopted over the years in ecological studies on insect populations. These include studies on insect mass rearing [14–16], pest control timing [17–18], host preference and fitness of insects [19–20] as well as the effects that temperature variations have on population dynamics [21–22].

It is essential to understand the population dynamics of a pest species before an effective integrated control program can be implemented [23–24]. Since life table parameters such as development, mortality, reproduction and a number of other elements are temperature-dependent [25–28], temperature is obviously the most crucial abiotic factor affecting the establishment, survival, reproduction and intrinsic rate of most insect populations [29–30]. For example, a previous study showed the relationship between survival rate and temperature on the development of *Plutella xylostella* for temperatures between 10°C to 35°C. Development occurred between 10°C to 32.5°C, but all individuals succumbed at 35°C [31]. Life table data offer a readily available means of tracking population growth as well as other changes [30], and are able to demonstrate relationships between temperature variations and population dynamics.

The traditional life table, which is based on only the female population, ignores the male population, different developmental stages and individual differences [11]. Population projects based on the age-stage, two-sex life table can eliminate many of the inherent errors characteristic of female-based life tables, since one of the main advantages of using the age-stage, two-sex life table is the ability to incorporate data from both sexes of a population [32–33]. Additionally, variations in pre-adult development time are precisely reflected in the survival and fecundity curves. Research projects comparing the demography of an insect species under different temperature conditions are usually regarded as an eco-friendly basis for developing control strategies for a given pest [9]. The aim of this study, therefore, was to amass as much data as possible on the biological and ecological parameters of *P*. *crisonalis* borer population growth under different temperature conditions, using data generated from the age-stage, two-sex life table obtained in this study, and to apply the results in making appropriate and effective strategic control decisions in future biocontrol programs.

## Materials and methods

### Insect culture

Larvae, pupae and adults of *P*. *crisonalis* were collected from experimental ponds on the Hunan Agriculture University campus (Changsha, Hunan Province, China; 28°110’, 113°40’). The larvae used in the various temperature studies had previously been laboratory bred for two complete generations in an artificial climate chamber kept under constant conditions (27 ± 1°C, 75 ± 20%RH and 10L: 14D). In order to maintain rearing conditions, larvae were provided with freshly excised leaves of water chestnut daily until larvae reached the pre-pupal stage. Following pupation, each pupa was kept in a separate rearing cage containing water chestnut leaves until eclosion. The rearing cages were covering with a mesh net to act as an oviposition substrate, and a clean cotton ball containing a 10% honey solution added daily as a nutritional supplement.

### Development time, oviposition period, fecundity and longevity

We placed 20 pairs of *P*. *crisonalis* adults in a separate rearing cage containing a potted water chestnut plant to obtain eggs of uniform maturity. The cage was covered with a mesh net to allow for adequate ventilation and to be used as an oviposition substrate. 80 to 160 eggs were collected and used to initiate each of the experimental treatments at 21, 24, 27, 30, 33 and 36°C; each egg was considered as a replicate. Water chestnut leaves were changed daily for the neonate larva. Each larva was considered as an individual replicate and was allowed to develop on a separate water chestnut plant until pupation. Pupae were removed and placed in individual glass tubes (2 cm diameter, 10 cm height) and covered with gauze. Moistened cotton balls had been placed in the bottom to retain moisture. Daily observations were conducted to observe larval mortality and to record each individual larval molt, pupation times and adult emergences. The combined larval and pupal development time was considered to be the total development time from egg to adult emergence, and defined as the pre-adult stage age. In addition, the adult longevities (from emergence to death for the males and females separately), entire lifespans (from egg to adults’ death), and mortality occurring in all stages were also recorded. The experiments were continued until the death of all individuals. After the pupae emerged as adults, males and females that had emerged on the same day were paired and each pair placed into a plastic oviposition container (13 cm diameter, 17 cm height). A single water chestnut plant, which had previously been planted in a disposable cup, was added to each of the plastic containers as an oviposition substrate. Each of the oviposition containers also contained a small cotton ball soaked with 10% honey solution for adults’ feeding. The numbers of eggs produced were recorded and the potted plant replaced daily. The adult pre-oviposition period (APOP: the period between the emergence of an adult female and her first oviposition), total pre-oviposition period (TPOP: the time interval from birth to the beginning of oviposition), oviposition period, daily fecundity, and total fecundity (number of eggs produced during the reproductive period) were also calculated using the experimental data.

### Data analysis

The age-stage, two-sex life table approach was used to analyze the raw life-history data for *P*. *crisonalis* [32, 34]. The age-stage–specific survival rate (*s*_*xj*_), (the probability of an individual of age *x* and stage *y* surviving to age *x*_*j*_ and stage *j*), was evaluated [35]. The age-stage-specific fecundity (*f*_*xj*_) (the daily number of eggs laid by an individual at age *x* and stage *j*), the age-specific fecundity curve (*m*_*x*_), the age-specific survival rate (*l*_*x*_) (the probability that a newly oviposited egg will survive to age *x*), and the population parameters were calculated accordingly [35]. The intrinsic rate of increase (*r*) was calculated on the basis of the Eule-Lotka equation as $\sum_{x=0}^{\infty }e^{-r\left(x+1\right)}l_{x}m_{x}=1$ [36]. The *GRR* was calculated as *GRR* = ∑*m*_*x*_. The finite rate of increase (*λ*) equaled *e*^*r*^. Net reproductive rate (*R*_*0*_) was measured as $\sum_{x=0}^{\infty }l_{x}m_{x}$. The mean generation time (*T*), defined as the time required for a population to increase to *R*_*0*_-fold of its population size at the stable stage distribution, was calculated using the formula *T* = (ln*R*_*0*_)/*r*. Finally, the means, standard errors and variances of the population parameters were estimated via the bootstrap technique [37], which is contained in the TWOSEX-MS Chart program. Sigma plot 12.5 was used to create graphs.

The raw life-history data for *P*. *crisonalis* obtained for each of the temperature regimes were entered separately into the Microsoft Excel 2013 program. The computer program, TWOSEX-MS Chart, for the age-stage two-sex life table analysis in VISUAL BASIC (version 6, service pack 6) for the Windows system, available on http://140.120.197.173/Ecology/ (National Chung Hsing University) and on http://nhsbig.inhs.uiuc.edu/wes/chi.html (Illinois Natural History Survey) [35], was used for raw data analysis. The program vastly simplifies the otherwise complicated and time consuming process of calculating the many parameters involved.

## Results

### Development time, adult longevity and lifespan

The mean durations of the total pre-adult stages of *P*. *crisonalis* at different temperature intervals between 21–36°C are given in Table 1. Because all eggs hatched on the same day in each of the different temperatures, except at 27 and 30°C, the standard error was 0. The durations of the egg stage did show significant difference (*P* < 0.05) at each of the six temperatures. The shortest developmental time for the egg stage was at 36°C and the longest at 21°C, with 21°C > 24°C > 27°C > 30°C > 33°C > 36°C in turn. The larval period also exhibited significant differences (*P* < 0.05) for the six different temperatures, with the shortest developmental time again occurring at 36°C and the longest at 21°C, with 21°C > 27°C > 24°C > 30°C > 33°C > 36°C in turn (Table 1). Although the longest pupal period occurred at 27°C, the shortest was at 30°C instead of 36°C, with 27°C > 21°C > 24°C > 36°C > 33°C > 30°C in turn. There was no significant difference (*P* > 0.05) between the 30°C and the 33°C treatments, nor were there significant differences (*P* > 0.05) between the 21, 24, 27 and 36°C treatments. The lengths of the total immature periods did show significant difference (*P* < 0.05) except at 24 and 27°C, with the shortest developmental time for the total immature period occurring at 36°C and the longest at 21°C, with 21°C > 27°C > 24°C > 30°C > 33°C > 36°C in turn. The highest (0.97) and lowest (0.65) mortality rates during the immature stages were found at 36°C and 27°C respectively, with 36°C > 21°C > 33°C > 30°C > 24°C > 27°C in turn. Except for 33 and 36°C, other rearing temperatures significant affected (*P* < 0.05) the entire lifespan of the males (Table 2). On the other hand, there were no significant differences (*P* > 0.05) in male adult longevity between 21 and 24 and 27°C (Table 2). The shortest total developmental time was at 36°C while the longest developmental time occurred at 21°C, for both female longevity and the female entire lifespan (Table 2).

**Table 1:** Developmental time (mean ± SE) and mortality of immature stages (days) of *P*. *crisonalis* reared at different temperatures under laboratory conditions.

	21°C		24°C		27°C		30°C		33°C		36°C		df	*F*	*P*
	n(N)	$\bar{x}+\text{SE}$	n(N)	$\bar{x}+\text{SE}$	n(N)	$\bar{x}+\text{SE}$	n(N)	$\bar{x}+\text{SE}$	n(N)	$\bar{x}+\text{SE}$	n(N)	$\bar{x}+\text{SE}$			
Egg	26(82)	7.00±0.00a	56(94)	6.00±0.00b	124(167)	4.17±0.03c	94(141)	3.19±0.04d	39(80)	3.00±0.00e	74(125)	2.00±0.00f	5,407	2166.11	< 0.0001
Larva	10(26)	24.60±1.25a	36(56)	14.78±0.28c	80(124)	16.71±0.22b	39(94)	13.28±0.41d	25(39)	11.60±0.24e	4(74)	5.00±0.58f	5,188	86.86	< 0.0001
Pupa	7(10)	5.86±0.38a	33(36)	5.67±0.14a	58(80)	6.02±0.12a	35(39)	3.71±0.15b	20(25)	3.85±0.13b	4(4)	5.00±0.63ab	5,151	41.12	< 0.0001
Total Immature Stages	7(7)	38.43±1.57a	33(33)	26.36±0.19b	58(58)	26.97±0.24b	35(35)	19.89±0.35c	20(20)	18.20±0.28d	4(4)	12.00±0.54e	5,151	225.99	< 0.0001
Mortality of immature stages	-	0.91±0.030a	-	0.65±0.049b	-	0.65±0.037b	-	0.75±0.036b	-	0.75±0.048b	-	0.97±0.015a	-	-	-

Note: N, total replicate number; n, effective replicate number; *$\bar{x}$*, mean value; SE, standard error; df, degree of freedom; *F*, value of Levene's Test; *P*, statistical significance. Means followed by different letters in the same row are significantly different by using paired bootstrap test based on the CI of difference. Standard errors were estimated by using 1,000,000 bootstrap resampling.

**Table 2:** Adult longevity and entire lifespan (days) of *P*. *crisonalis* reared at different temperatures under laboratory conditions.

	21°C		24°C		27°C		30°C		33°C		36°C		df	*F*	*P*
	n	$\bar{x}+\text{SE}$	n	$\bar{x}+\text{SE}$	n	$\bar{x}+\text{SE}$	n	$\bar{x}+\text{SE}$	N	$\bar{x}+\text{SE}$	n	$\bar{x}+\text{SE}$			
Male adult	5	6.20±0.99a	15	7.20±0.54a	29	4.17±0.24ab	21	3.14±0.14b	13	3.15±0.15b	2	2.00±0.68b	5,79	23.66	< 0.0001
Female adult	2	4.50±0.38a	18	4.28±0.19a	29	4.34±0.23a	14	3.50±0.27b	7	3.14±0.14b	2	3.00±0.75ab	5,66	2.97	0.018
Male entire lifespan	5	43.60±3.63a	15	33.00±0.46b	29	30.79±0.42c	21	22.76±0.39d	13	21.23±0.36e	2	14.00±4.81e	5,79	164.85	< 0.0001
Female entire lifespan	2	45.50±1.14a	18	31.11±0.33b	29	31.66±0.42b	14	23.79±0.82c	7	21.57±0.52d	2	15.00±0.00e	5,66	82.80	< 0.0001

Note: n, effective replicate number; *$\bar{x}$*, mean value; SE, standard error; df, degree of freedom; *F*, value of Levene's Test; *P*, statistical significance. Means followed by different letters in the same row are significantly different by using paired bootstrap test based on the CI of difference.

### Oviposition period and fecundity

The APOP proved to be longest at 24°C, with 24°C > 27°C > 30°C = 33°C > 36°C > 21°C in turn, while the TPOP was the longest at 21°C, with 21°C > 27°C > 24°C > 30°C > 33°C > 36°C in turn (Table 3). Females reared at 24°C had the highest fecundity, while the lowest fecundity occurred at 33°C, with 24°C > 27°C > 30°C > 36°C = 21°C > 33°C in turn (Table 3). The number of oviposition days for females decreased from 24°C to 30°C, with similar results found at 21°C, 33°C and 36°C, with 24°C > 27°C > 30°C > 33°C = 36°C = 21°C in turn (Table 3).

**Table 3:** Adult Preoviposition Period (APOP), Total Preoviposition Period (TPOP), oviposition period, and fecundity of *P*. *crisonalis* reared at different temperature.

	21°C		24°C		27°C		30°C		33°C		36°C		df	*F*	*P*
	n	*$\bar{x}+\text{SE}$*	N	$\bar{x}+\text{SE}$	n	$\bar{x}+\text{SE}$	n	$\bar{x}+\text{SE}$	N	$\bar{x}+\text{SE}$	n	$\bar{x}+\text{SE}$			
APOP	2	1.00±0.00b	17	2.06±0.06a	29	2.03±0.08a	12	2.00±0.00a	2	2.00±0.00a	2	1.50±0.38ab	5,58	4.67	0.00197
TPOP	2	42.00±1.52a	17	28.88±0.22b	29	29.34±0.33b	12	22.50±0.73c	2	19.00±0.76d	2	13.50±0.38e	5,58	87.60	< 0.0001
Oviposition period	2	1.00±0.00b	17	2.29±0.19a	29	2.28±0.20a	12	1.75±0.25a	2	1.00±0.00b	2	1.00±0.00b	5,66	6.01	0.00012
Fecundity (Eggs/female)	2	7.00±0.76c	18	349.28±45.76a	29	293.83±41.62a	14	164.86±45.15b	7	3.00±1.70d	2	7.00±0.76c	5,66	5.35	0.00035

Note: APOP, adult preovipositional period; TPOP, total preovipositional period (from egg to first oviposition); n, effective replicate number; $\bar{x}$, mean value; SE, standard error; df, degree of freedom; *F*, value of Levene's Test; *P*, statistical significance. Means followed by different letters in the same row are significantly different by using paired bootstrap test based on the CI of difference.

### Life table analysis

**Fig 1:**
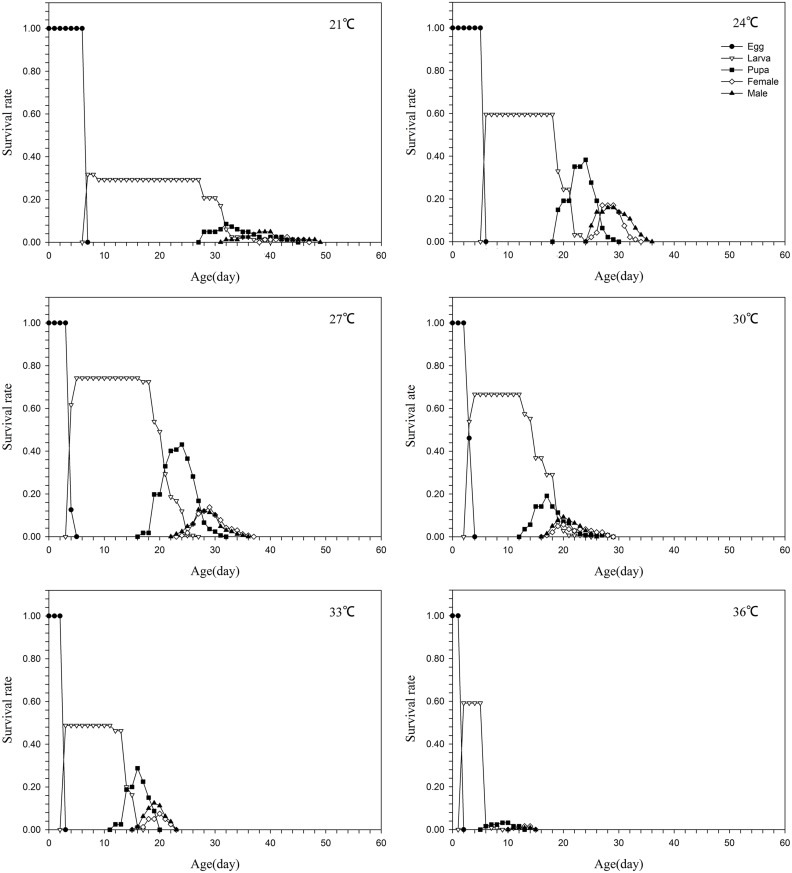
Age-stage-specific survival rate of *Parapoynx crisonalis*.

**Fig 2:**
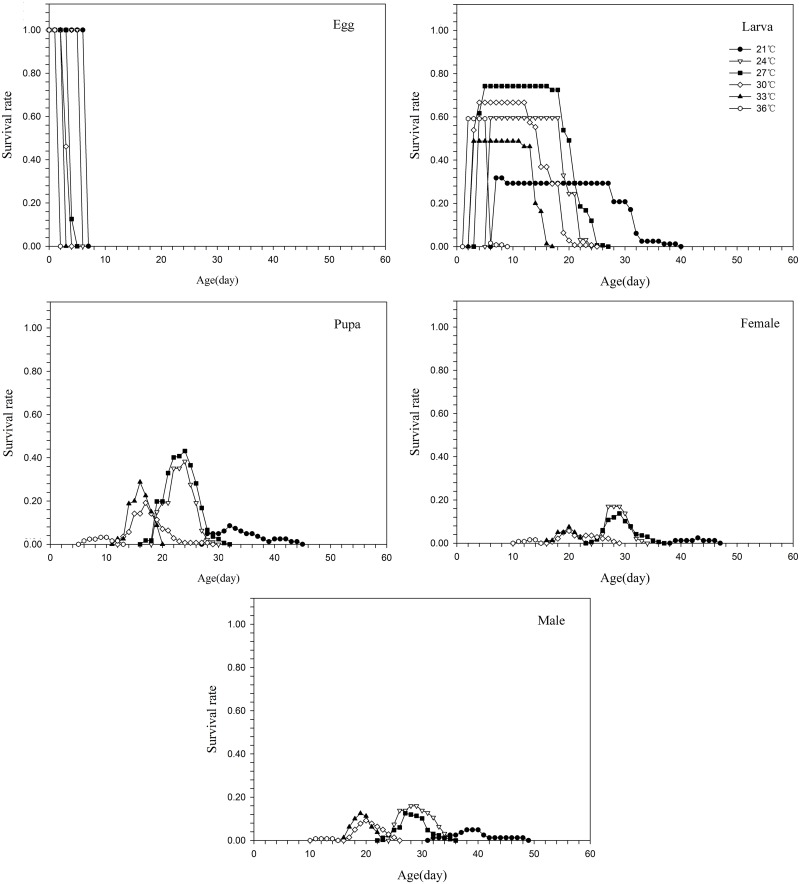
Age-temperature-specific survival rate of *Parapoynx crisonalis*.

**Fig 3:**
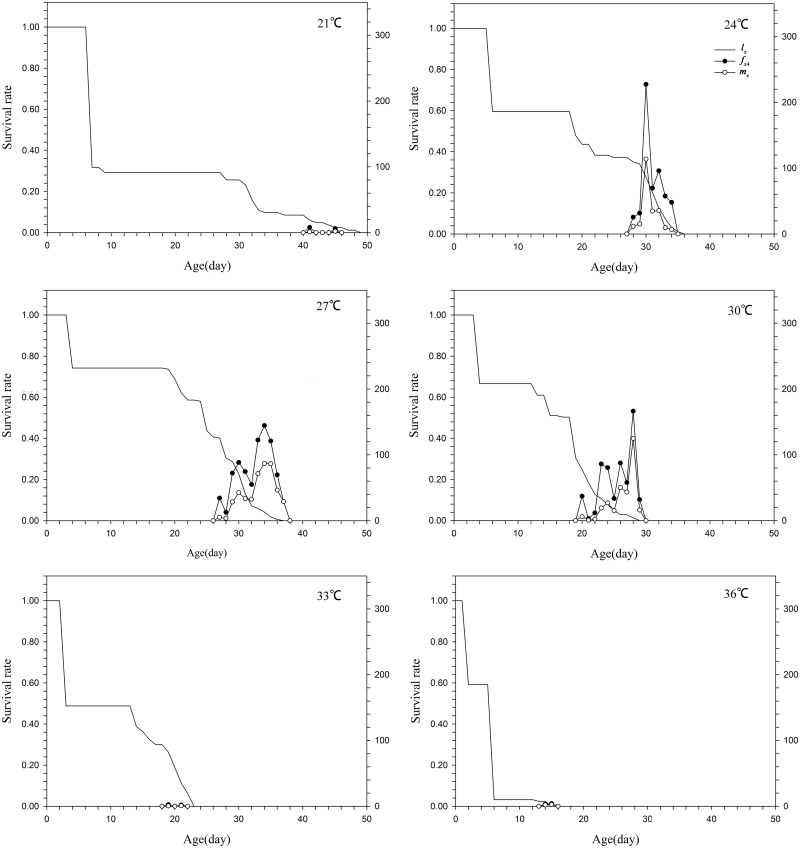
Age-specific survival rate, age-stage-specific fecundity and age-specific fecundity of *Parapoynx crisonalis*.

The lowest survival rate of the eggs occurred at 21°C and the highest at 30°C (Figs 1 & 2), while the lowest survival rates for the larval and the pupal stages were at 33°C. The highest survival rates for the larval and the pupal stages were at 27°C and 24°C, respectively. The specific survival rates of larvae and pupae were all very low at 21°C and 36°C (Figs 1 & 2). The age-stage-specific fecundity (*f*_*x*_) peak was at 24°C and the age-specific fecundity (*l*_*x*_) peak was at 30°C. The *f*_*x*_ and *m*_*x*_ values were all very low at 21°C, 33°C and 36°C (Fig 3).

The highest value for the net reproductive rate (*R*_0_) was 66.88, occurring at 24°C, while the lowest (0.11) was at 36°C, with 24°C > 27°C > 30°C > 33°C > 21°C > 36°C in turn (Table 4). The highest *GRR* value (464.66) was found at 27°C; while the lowest, at 33°C, was only 1.15, with 27°C > 30°C > 24°C > 21°C > 36°C > 33°C in turn. The intrinsic rates of increase (*r*) reached their highest value (0.14) at a temperature of 24°C, and their lowest (-0.15) at 36°C, with 24°C > 27°C > 30°C > 21°C > 33°C > 36°C in turn. The highest values for the finite rate (λ) of increase were 1.15 at 24°C, while the lowest was 0.86 at 36°C, with 24°C > 27°C > 30°C > 21°C > 33°C > 36°C in turn. The longest mean generation time was 42.80 days at 21°C, compared to the shortest of 14.59 days at 36°C, with 21°C > 27°C > 24°C > 30°C > 33°C > 36°C in turn.

**Table 4:** Life table parameters of *P*. *crisonalis* reared at different temperatures.

	21°C	24°C	27°C	30°C	33°C	36°C
	$\bar{x}+\text{SE}$	$\bar{x}+\text{SE}$	$\bar{x}+\text{SE}$	$\bar{x}+\text{SE}$	$\bar{x}+\text{SE}$	$\bar{x}+\text{SE}$
*R*_0_	0.17±0.108c	66.88±16.533a	51.02±11.136a	16.37±5.999b	0.26±0.164c	0.11±0.071c
*GRR*	3.14±1.930bc	227.12±52.212a	464.66±102.679a	304.03±88.509a	1.15±0.767c	4.67±1.766b
*r*	-0.041±0.013b	0.14±0.009a	0.13±0.008a	0.11±0.018a	-0.07±0.028bc	-0.15±0.040c
λ	0.96±0.012b	1.15±0.010a	1.14±0.009a	1.12±0.019a	0.94±0.026bc	0.86±0.034c
*T*	42.80±1.521a	30.16±0.264b	30.86±0.49b	24.47±0.999c	20.08±0.759d	14.59±0.381e

Note: *R*_0,_ net reproductive rate; *GRR*, gross reproductive rate; *r*, intrinsic rate of increase; λ, finite rate of increase; *T*, mean generation time; $\bar{x}$, mean value; SE, standard error. Means followed by different letters in the same row are significantly different by using paired bootstrap test based on the CI of difference. Standard errors were estimated by using 1,000,000 bootstrap resampling

## Discussion

Because the susceptibility of insects to various environmental factors, pesticides, natural enemies, etc., often differs depending on their developmental stage, information regarding the population stage structure is critical in achieving effective pest management [38].

We used an array of six different rearing temperatures (21, 24, 27, 30, 33 and 36°C) in the present study to examine the effects of varying temperatures on *P*. *crisonalis*. The temperature had a significant influence on the entire lifespan of males and females. The male entire lifespan gradually decreased as temperatures increased from 21 to 36°C. The female lifespan also decreased with increasing temperature except at 27°C, where it was slightly longer than at 24°C. The fecundity of *P*. *crisonalis* proved to be a vital parameter, graphically demonstrating the critical role that temperature plays in the population dynamics of the species. Decreases in female fecundity have been shown to occur in various other insect species following temperature increases [39–40]. *Parapoynx crisonalis* females were capable of reproducing at all temperatures (21–36°C) (Fig 3). The fecundity between 24°C and 30°C gradually decreased, whereas the groups reared at 21, 30 and 36°C exhibited low fecundity compared to the other three groups (Table 3). Female fecundity was positively correlated with the oviposition period. The variation trend of the oviposition period was similar to that of fecundity.

The *r* value was introduced as a useful concept for studying insect populations by Huang and Chi [41]. According to life table theory a population was increasing only when *R*_*O*_ > 1 and *r* > 0 [42]. In the present study, *Ro* > 1 and *r* > 0 only from 24°C to 30°C, indicating that the *P*. *crisonalis* population increased in this temperature range; the *P*. *crisonalis* population could not increase, however when *r* < 0 at 21, 33 and 36°C (Table 4). The bootstrap technique was used to estimate the means and variance of the population parameters. The highest *R*_*0*_ value for *P*. *crisonalis* (66.88) was found in the 24°C experimental group. Among the other population parameters, the intrinsic rate of increase (*r*) is a critical demographic element for determining levels of environment resistance to insects [43]. Contrasting the *R*_*0*_ and *r* values invariably yields considerable insight beyond that obtainable from independent analysis of individual life-history parameters [44]. Conversely, the lower life table values found in the 21°C, 33°C and 36°C temperature regimes revealed that these conditions were unfavorable to the pest.

## Supporting information

S1 Data Set Fig 1 Age-stage-specific survival rate of *Parapoynx crisonalis*. (DOCX)

S2 Data Set Fig 2 Age-temperature-specific survival rate of *Parapoynx crisonalis*. (DOCX)

S3 Data Set Fig 3 Age-specific survival rate, age-stage-specific fecundity and age-specific fecundity of *Parapoynx crisonalis*. (DOCX)
