# Supplementary material for: Age-stage, two-sex life table of Parapoynx crisonalis (Lepidoptera: Pyralidae) at different temperatures
Source: PLoS One. 2017 Mar 6;12(3):e0173380. doi: 10.1371/journal.pone.0173380 (PMC5338836; doi:10.1371/journal.pone.0173380)
Supplement: S1 Data Set — (DOCX) [file pone.0173380.s001.docx]

**S1 Data Set. Fig. 1 Age-stage-specific survival rate of *Parapoynx crisonalis*.**

**21℃**

| Insect stage   \| Age  Survival rate \| \| --- \| \| | Egg | Larva | Pupa | Female | Male |
| --- | --- | --- | --- | --- | --- | --- |
| 0 | 1 | 0 | 0 | 0 | 0 |
| 1 | 1 | 0 | 0 | 0 | 0 |
| 2 | 1 | 0 | 0 | 0 | 0 |
| 3 | 1 | 0 | 0 | 0 | 0 |
| 4 | 1 | 0 | 0 | 0 | 0 |
| 5 | 1 | 0 | 0 | 0 | 0 |
| 6 | 1 | 0 | 0 | 0 | 0 |
| 7 | 0 | 0.317073171 | 0 | 0 | 0 |
| 8 | 0 | 0.317073171 | 0 | 0 | 0 |
| 9 | 0 | 0.292682927 | 0 | 0 | 0 |
| 10 | 0 | 0.292682927 | 0 | 0 | 0 |
| 11 | 0 | 0.292682927 | 0 | 0 | 0 |
| 12 | 0 | 0.292682927 | 0 | 0 | 0 |
| 13 | 0 | 0.292682927 | 0 | 0 | 0 |
| 14 | 0 | 0.292682927 | 0 | 0 | 0 |
| 15 | 0 | 0.292682927 | 0 | 0 | 0 |
| 16 | 0 | 0.292682927 | 0 | 0 | 0 |
| 17 | 0 | 0.292682927 | 0 | 0 | 0 |
| 18 | 0 | 0.292682927 | 0 | 0 | 0 |
| 19 | 0 | 0.292682927 | 0 | 0 | 0 |
| 20 | 0 | 0.292682927 | 0 | 0 | 0 |
| 21 | 0 | 0.292682927 | 0 | 0 | 0 |
| 22 | 0 | 0.292682927 | 0 | 0 | 0 |
| 23 | 0 | 0.292682927 | 0 | 0 | 0 |
| 24 | 0 | 0.292682927 | 0 | 0 | 0 |
| 25 | 0 | 0.292682927 | 0 | 0 | 0 |
| 26 | 0 | 0.292682927 | 0 | 0 | 0 |
| 27 | 0 | 0.292682927 | 0 | 0 | 0 |
| 28 | 0 | 0.207317073 | 4.88E-02 | 0 | 0 |
| 29 | 0 | 0.207317073 | 4.88E-02 | 0 | 0 |
| 30 | 0 | 0.207317073 | 4.88E-02 | 0 | 0 |
| 31 | 0 | 0.170731707 | 6.10E-02 | 0 | 0 |
| 32 | 0 | 6.10E-02 | 8.54E-02 | 0 | 1.22E-02 |
| 33 | 0 | 0.024390244 | 7.32E-02 | 0 | 1.22E-02 |
| 34 | 0 | 0.024390244 | 6.10E-02 | 0 | 1.22E-02 |
| 35 | 0 | 0.024390244 | 4.88E-02 | 0 | 0.024390244 |
| 36 | 0 | 0.024390244 | 4.88E-02 | 0 | 0.024390244 |
| 37 | 0 | 1.22E-02 | 3.66E-02 | 0 | 3.66E-02 |
| 38 | 0 | 1.22E-02 | 0.024390244 | 0 | 4.88E-02 |
| 39 | 0 | 1.22E-02 | 1.22E-02 | 1.22E-02 | 4.88E-02 |
| 40 | 0 | 0 | 0.024390244 | 1.22E-02 | 4.88E-02 |
| 41 | 0 | 0 | 0.024390244 | 1.22E-02 | 0.024390244 |
| 42 | 0 | 0 | 0.024390244 | 1.22E-02 | 1.22E-02 |
| 43 | 0 | 0 | 1.22E-02 | 0.024390244 | 1.22E-02 |
| 44 | 0 | 0 | 1.22E-02 | 1.22E-02 | 1.22E-02 |
| 45 | 0 | 0 | 0 | 1.22E-02 | 1.22E-02 |
| 46 | 0 | 0 | 0 | 1.22E-02 | 1.22E-02 |
| 47 | 0 | 0 | 0 | 0 | 1.22E-02 |
| 48 | 0 | 0 | 0 | 0 | 1.22E-02 |
| 49 | 0 | 0 | 0 | 0 | 0 |

**24℃**

| Insect stage   \| Age  Survival rate \| \| --- \| \| | Egg | Larva | Pupa | Female | Male |
| --- | --- | --- | --- | --- | --- | --- |
| 0 | 1 | 0 | 0 | 0 | 0 |
| 1 | 1 | 0 | 0 | 0 | 0 |
| 2 | 1 | 0 | 0 | 0 | 0 |
| 3 | 1 | 0 | 0 | 0 | 0 |
| 4 | 1 | 0 | 0 | 0 | 0 |
| 5 | 1 | 0 | 0 | 0 | 0 |
| 6 | 0 | 0.595744681 | 0 | 0 | 0 |
| 7 | 0 | 0.595744681 | 0 | 0 | 0 |
| 8 | 0 | 0.595744681 | 0 | 0 | 0 |
| 9 | 0 | 0.595744681 | 0 | 0 | 0 |
| 10 | 0 | 0.595744681 | 0 | 0 | 0 |
| 11 | 0 | 0.595744681 | 0 | 0 | 0 |
| 12 | 0 | 0.595744681 | 0 | 0 | 0 |
| 13 | 0 | 0.595744681 | 0 | 0 | 0 |
| 14 | 0 | 0.595744681 | 0 | 0 | 0 |
| 15 | 0 | 0.595744681 | 0 | 0 | 0 |
| 16 | 0 | 0.595744681 | 0 | 0 | 0 |
| 17 | 0 | 0.595744681 | 0 | 0 | 0 |
| 18 | 0 | 0.595744681 | 0 | 0 | 0 |
| 19 | 0 | 0.329787234 | 0.14893617 | 0 | 0 |
| 20 | 0 | 0.244680851 | 0.191489362 | 0 | 0 |
| 21 | 0 | 0.244680851 | 0.191489362 | 0 | 0 |
| 22 | 0 | 3.19E-02 | 0.35106383 | 0 | 0 |
| 23 | 0 | 3.19E-02 | 0.35106383 | 0 | 0 |
| 24 | 0 | 0 | 0.382978723 | 0 | 0 |
| 25 | 0 | 0 | 0.276595745 | 2.13E-02 | 0.074468085 |
| 26 | 0 | 0 | 0.191489362 | 4.26E-02 | 0.138297872 |
| 27 | 0 | 0 | 6.38E-02 | 0.170212766 | 0.138297872 |
| 28 | 0 | 0 | 2.13E-02 | 0.170212766 | 0.159574468 |
| 29 | 0 | 0 | 1.06E-02 | 0.170212766 | 0.159574468 |
| 30 | 0 | 0 | 0 | 0.138297872 | 0.138297872 |
| 31 | 0 | 0 | 0 | 0.074468085 | 0.127659574 |
| 32 | 0 | 0 | 0 | 2.13E-02 | 0.106382979 |
| 33 | 0 | 0 | 0 | 1.06E-02 | 6.38E-02 |
| 34 | 0 | 0 | 0 | 0 | 3.19E-02 |
| 35 | 0 | 0 | 0 | 0 | 1.06E-02 |
| 36 | 0 | 0 | 0 | 0 | 0 |

**27℃**

| Insect stage   \| Age  Survival rate \| \| --- \| \| | Egg | Larva | Pupa | Female | Male |
| --- | --- | --- | --- | --- | --- | --- |
| 0 | 1 | 0 | 0 | 0 | 0 |
| 1 | 1 | 0 | 0 | 0 | 0 |
| 2 | 1 | 0 | 0 | 0 | 0 |
| 3 | 1 | 0 | 0 | 0 | 0 |
| 4 | 0.125748503 | 0.616766467 | 0 | 0 | 0 |
| 5 | 0 | 0.74251497 | 0 | 0 | 0 |
| 6 | 0 | 0.74251497 | 0 | 0 | 0 |
| 7 | 0 | 0.74251497 | 0 | 0 | 0 |
| 8 | 0 | 0.74251497 | 0 | 0 | 0 |
| 9 | 0 | 0.74251497 | 0 | 0 | 0 |
| 10 | 0 | 0.74251497 | 0 | 0 | 0 |
| 11 | 0 | 0.74251497 | 0 | 0 | 0 |
| 12 | 0 | 0.74251497 | 0 | 0 | 0 |
| 13 | 0 | 0.74251497 | 0 | 0 | 0 |
| 14 | 0 | 0.74251497 | 0 | 0 | 0 |
| 15 | 0 | 0.74251497 | 0 | 0 | 0 |
| 16 | 0 | 0.74251497 | 0 | 0 | 0 |
| 17 | 0 | 0.724550898 | 1.80E-02 | 0 | 0 |
| 18 | 0 | 0.724550898 | 1.80E-02 | 0 | 0 |
| 19 | 0 | 0.538922156 | 0.19760479 | 0 | 0 |
| 20 | 0 | 0.491017964 | 0.19760479 | 0 | 0 |
| 21 | 0 | 0.293413174 | 0.329341317 | 0 | 0 |
| 22 | 0 | 0.185628743 | 0.401197605 | 0 | 0 |
| 23 | 0 | 0.167664671 | 0.407185629 | 0 | 1.20E-02 |
| 24 | 0 | 0.119760479 | 0.431137725 | 5.99E-03 | 2.40E-02 |
| 25 | 0 | 5.99E-03 | 0.365269461 | 1.80E-02 | 4.79E-02 |
| 26 | 0 | 5.99E-03 | 0.281437126 | 5.99E-02 | 5.99E-02 |
| 27 | 0 | 0 | 0.167664671 | 0.107784431 | 0.125748503 |
| 28 | 0 | 0 | 6.59E-02 | 0.119760479 | 0.119760479 |
| 29 | 0 | 0 | 3.59E-02 | 0.137724551 | 0.113772455 |
| 30 | 0 | 0 | 2.40E-02 | 0.101796407 | 0.101796407 |
| 31 | 0 | 0 | 5.99E-03 | 7.78E-02 | 4.79E-02 |
| 32 | 0 | 0 | 0 | 4.19E-02 | 0.02994012 |
| 33 | 0 | 0 | 0 | 3.59E-02 | 2.40E-02 |
| 34 | 0 | 0 | 0 | 0.02994012 | 1.20E-02 |
| 35 | 0 | 0 | 0 | 1.20E-02 | 5.99E-03 |
| 36 | 0 | 0 | 0 | 5.99E-03 | 0 |
| 37 | 0 | 0 | 0 | 0 | 0 |

**30℃**

| Insect stage   \| Age  Survival rate \| \| --- \| \| | Egg | Larva | Pupa | Female | Male |
| --- | --- | --- | --- | --- | --- | --- |
| 0 | 1 | 0 | 0 | 0 | 0 |
| 1 | 1 | 0 | 0 | 0 | 0 |
| 2 | 1 | 0 | 0 | 0 | 0 |
| 3 | 0.460992908 | 0.539007092 | 0 | 0 | 0 |
| 4 | 0 | 0.666666667 | 0 | 0 | 0 |
| 5 | 0 | 0.666666667 | 0 | 0 | 0 |
| 6 | 0 | 0.666666667 | 0 | 0 | 0 |
| 7 | 0 | 0.666666667 | 0 | 0 | 0 |
| 8 | 0 | 0.666666667 | 0 | 0 | 0 |
| 9 | 0 | 0.666666667 | 0 | 0 | 0 |
| 10 | 0 | 0.666666667 | 0 | 0 | 0 |
| 11 | 0 | 0.666666667 | 0 | 0 | 0 |
| 12 | 0 | 0.666666667 | 0 | 0 | 0 |
| 13 | 0 | 0.574468085 | 3.55E-02 | 0 | 0 |
| 14 | 0 | 0.553191489 | 5.67E-02 | 0 | 0 |
| 15 | 0 | 0.368794326 | 0.141843972 | 0 | 0 |
| 16 | 0 | 0.368794326 | 0.141843972 | 0 | 0 |
| 17 | 0 | 0.290780142 | 0.191489362 | 7.09E-03 | 1.42E-02 |
| 18 | 0 | 0.290780142 | 0.141843972 | 2.13E-02 | 0.04964539 |
| 19 | 0 | 6.38E-02 | 0.113475177 | 0.04964539 | 7.80E-02 |
| 20 | 0 | 2.84E-02 | 7.09E-02 | 5.67E-02 | 9.22E-02 |
| 21 | 0 | 7.09E-03 | 6.38E-02 | 3.55E-02 | 7.80E-02 |
| 22 | 0 | 7.09E-03 | 2.84E-02 | 2.84E-02 | 6.38E-02 |
| 23 | 0 | 7.09E-03 | 1.42E-02 | 3.55E-02 | 0.04964539 |
| 24 | 0 | 7.09E-03 | 7.09E-03 | 3.55E-02 | 2.84E-02 |
| 25 | 0 | 0 | 7.09E-03 | 2.84E-02 | 1.42E-02 |
| 26 | 0 | 0 | 7.09E-03 | 2.13E-02 | 0 |
| 27 | 0 | 0 | 7.09E-03 | 2.13E-02 | 0 |
| 28 | 0 | 0 | 7.09E-03 | 7.09E-03 | 0 |
| 29 | 0 | 0 | 0 | 0 | 0 |

**33℃**

| Insect stage   \| Age  Survival rate \| \| --- \| \| | Egg | Larva | Pupa | Female | Male |
| --- | --- | --- | --- | --- | --- | --- |
| 0 | 1 | 0 | 0 | 0 | 0 |
| 1 | 1 | 0 | 0 | 0 | 0 |
| 2 | 1 | 0 | 0 | 0 | 0 |
| 3 | 0 | 0.4875 | 0 | 0 | 0 |
| 4 | 0 | 0.4875 | 0 | 0 | 0 |
| 5 | 0 | 0.4875 | 0 | 0 | 0 |
| 6 | 0 | 0.4875 | 0 | 0 | 0 |
| 7 | 0 | 0.4875 | 0 | 0 | 0 |
| 8 | 0 | 0.4875 | 0 | 0 | 0 |
| 9 | 0 | 0.4875 | 0 | 0 | 0 |
| 10 | 0 | 0.4875 | 0 | 0 | 0 |
| 11 | 0 | 0.4875 | 0 | 0 | 0 |
| 12 | 0 | 0.4625 | 0.025 | 0 | 0 |
| 13 | 0 | 0.4625 | 0.025 | 0 | 0 |
| 14 | 0 | 0.2 | 0.1875 | 0 | 0 |
| 15 | 0 | 0.1625 | 0.2 | 0 | 0 |
| 16 | 0 | 0.0125 | 0.2875 | 0.0125 | 0.0125 |
| 17 | 0 | 0 | 0.225 | 0.0125 | 0.0625 |
| 18 | 0 | 0 | 0.15 | 0.05 | 0.1 |
| 19 | 0 | 0 | 0.0875 | 0.05 | 0.125 |
| 20 | 0 | 0 | 0 | 0.075 | 0.1125 |
| 21 | 0 | 0 | 0 | 0.05 | 0.0625 |
| 22 | 0 | 0 | 0 | 0.025 | 0.0375 |
| 23 | 0 | 0 | 0 | 0 | 0 |

**36℃**

| Insect stage   \| Age  Survival rate \| \| --- \| \| | Egg | Larva | Pupa | Female | Male |
| --- | --- | --- | --- | --- | --- | --- |
| 0 | 1 | 0 | 0 | 0 | 0 |
| 1 | 1 | 0 | 0 | 0 | 0 |
| 2 | 0 | 0.592 | 0 | 0 | 0 |
| 3 | 0 | 0.592 | 0 | 0 | 0 |
| 4 | 0 | 0.592 | 0 | 0 | 0 |
| 5 | 0 | 0.592 | 0 | 0 | 0 |
| 6 | 0 | 0.016 | 0.016 | 0 | 0 |
| 7 | 0 | 0.008 | 0.024 | 0 | 0 |
| 8 | 0 | 0.008 | 0.024 | 0 | 0 |
| 9 | 0 | 0 | 0.032 | 0 | 0 |
| 10 | 0 | 0 | 0.032 | 0 | 0 |
| 11 | 0 | 0 | 0.016 | 0.008 | 0.008 |
| 12 | 0 | 0 | 0.016 | 0.008 | 0.008 |
| 13 | 0 | 0 | 0 | 0.016 | 0.008 |
| 14 | 0 | 0 | 0 | 0.016 | 0.008 |
| 15 | 0 | 0 | 0 | 0 | 0 |
