# Supplementary material for: Age-stage, two-sex life table of Parapoynx crisonalis (Lepidoptera: Pyralidae) at different temperatures
Source: PLoS One. 2017 Mar 6;12(3):e0173380. doi: 10.1371/journal.pone.0173380 (PMC5338836; doi:10.1371/journal.pone.0173380)
Supplement: S2 Data Set — (DOCX) [file pone.0173380.s002.docx]

**S2 Data Set. Fig. 2 Age-temperature-specific survival rate of *Parapoynx crisonalis*.**

**Egg**

| Temperature   \| Age  Survival rate \| \| --- \| \| | | 21℃ | | 24℃ | 27℃ | | | 30℃ | | 33℃ | | 36℃ |  |
| --- | --- | --- | --- | --- | --- | --- | --- | --- | --- | --- | --- | --- | --- | --- |
| 0 | 1 | | 1 | | | 1 | | 1 | | 1 | 1 | |  |
| 1 | 1 | | 1 | | | 1 | | 1 | | 1 | 1 | |  |
| 2 | 1 | | 1 | | | 1 | | 1 | | 1 | 0 | |  |
| 3 | 1 | | 1 | | | 1 | | 0.460992908 | | 0 | 0 | |  |
| 4 | 1 | | 1 | | | 0.125748503 | | 0 | | 0 | 0 | |  |
| 5 | 1 | | 1 | | | 0 | | 0 | | 0 | 0 | |  |
| 6 | 1 | | 0 | | | 0 | | 0 | | 0 | 0 | |  |
| 7 | 0 | | 0 | | | 0 | | 0 | | 0 | 0 | |  |
| 8 | 0 | | 0 | | | 0 | | 0 | | 0 | 0 | |  |
| 9 | 0 | | 0 | | | 0 | | 0 | | 0 | 0 | |  |
| 10 | 0 | | 0 | | | 0 | | 0 | | 0 | 0 | |  |
| 11 | 0 | | 0 | | | 0 | | 0 | | 0 | 0 | |  |
| 12 | 0 | | 0 | | | 0 | | 0 | | 0 | 0 | |  |
| 13 | 0 | | 0 | | | 0 | | 0 | | 0 | 0 | |  |
| 14 | 0 | | 0 | | | 0 | | 0 | | 0 | 0 | |  |
| 15 | 0 | | 0 | | | 0 | | 0 | | 0 | 0 | |  |
| 16 | 0 | | 0 | | | 0 | | 0 | | 0 |  |  |  |
| 17 | 0 | | 0 | | | 0 | | 0 | | 0 |  |  |  |
| 18 | 0 | | 0 | | | 0 | | 0 | | 0 |  |  |  |
| 19 | 0 | | 0 | | | 0 | | 0 | | 0 |  |  |  |
| 20 | 0 | | 0 | | | 0 | | 0 | | 0 |  |  |  |
| 21 | 0 | | 0 | | | 0 | | 0 | | 0 |  |  |  |
| 22 | 0 | | 0 | | | 0 | | 0 | | 0 |  |  |  |
| 23 | 0 | | 0 | | | 0 | | 0 | | 0 |  |  |  |
| 24 | 0 | | 0 | | | 0 | | 0 | |  |  |  |  |
| 25 | 0 | | 0 | | | 0 | | 0 | |  |  |  |  |
| 26 | 0 | | 0 | | | 0 | | 0 | |  |  |  |  |
| 27 | 0 | | 0 | | | 0 | | 0 | |  |  |  |  |
| 28 | 0 | | 0 | | | 0 | | 0 | |  |  |  |  |
| 29 | 0 | | 0 | | | 0 | | 0 | |  |  |  |  |
| 30 | 0 | | 0 | | | 0 | |  |  |  |  |  |  |
| 31 | 0 | | 0 | | | 0 | |  |  |  |  |  |  |
| 32 | 0 | | 0 | | | 0 | |  |  |  |  |  |  |
| 33 | 0 | | 0 | | | 0 | |  |  |  |  |  |  |
| 34 | 0 | | 0 | | | 0 | |  |  |  |  |  |  |
| 35 | 0 | | 0 | | | 0 | |  |  |  |  |  |  |
| 36 | 0 | | 0 | | | 0 | |  |  |  |  |  |  |
| 37 | 0 | |  | | | | 0 | |  |  |  |  |  |
| 38 | 0 | |  |  |  |  |  |  |  |  |  |  |  |
| 39 | 0 | |  |  |  |  |  |  |  |  |  |  |  |
| 40 | 0 | |  |  |  |  |  |  |  |  |  |  |  |
| 41 | 0 | |  |  |  |  |  |  |  |  |  |  |  |
| 42 | 0 | |  |  |  |  |  |  |  |  |  |  |  |
| 43 | 0 | |  |  |  |  |  |  |  |  |  |  |  |
| 44 | 0 | |  |  |  |  |  |  |  |  |  |  |  |
| 45 | 0 | |  |  |  |  |  |  |  |  |  |  |  |
| 46 | 0 | |  |  |  |  |  |  |  |  |  |  |  |
| 47 | 0 | |  |  |  |  |  |  |  |  |  |  |  |
| 48 | 0 | |  |  |  |  |  |  |  |  |  |  |  |
| 49 | 0 | |  |  |  |  |  |  |  |  |  |  |  |

**Larva**

| Temperature   \| Age  Survival rate \| \| --- \| \| | | 21℃ | | 24℃ | 27℃ | | | | | 30℃ | 33℃ | | | 36℃ |  |  |
| --- | --- | --- | --- | --- | --- | --- | --- | --- | --- | --- | --- | --- | --- | --- | --- | --- | --- |
| 0 | 0 | | 0 | | | | 0 | | 0 | | | 0 | 0 | | | |
| 1 | 0 | | 0 | | | | 0 | | 0 | | | 0 | 0 | | | |
| 2 | 0 | | 0 | | | | 0 | | 0 | | | 0 | 0.592 | | | |
| 3 | 0 | | 0 | | | | 0 | | 0.539007092 | | | 0.4875 | 0.592 | | | |
| 4 | 0 | | 0 | | | | 0.616766467 | | 0.666666667 | | | 0.4875 | 0.592 | | | |
| 5 | 0 | | 0 | | | | 0.74251497 | | 0.666666667 | | | 0.4875 | 0.592 | | | |
| 6 | 0 | | 0.595744681 | | | | 0.74251497 | | 0.666666667 | | | 0.4875 | 0.016 | | | |
| 7 | 0.317073171 | | 0.595744681 | | | | 0.74251497 | | 0.666666667 | | | 0.4875 | 0.008 | | | |
| 8 | 0.317073171 | | 0.595744681 | | | | 0.74251497 | | 0.666666667 | | | 0.4875 | 0.008 | | | |
| 9 | 0.292682927 | | 0.595744681 | | | | 0.74251497 | | 0.666666667 | | | 0.4875 | 0 | | | |
| 10 | 0.292682927 | | 0.595744681 | | | | 0.74251497 | | 0.666666667 | | | 0.4875 | 0 | | | |
| 11 | 0.292682927 | | 0.595744681 | | | | 0.74251497 | | 0.666666667 | | | 0.4875 | 0 | | | |
| 12 | 0.292682927 | | 0.595744681 | | | | 0.74251497 | | 0.666666667 | | | 0.4625 | 0 | | | |
| 13 | 0.292682927 | | 0.595744681 | | | | 0.74251497 | | 0.574468085 | | | 0.4625 | 0 | | | |
| 14 | 0.292682927 | | 0.595744681 | | | | 0.74251497 | | 0.553191489 | | | 0.2 | 0 | | | |
| 15 | 0.292682927 | | 0.595744681 | | | | 0.74251497 | | 0.368794326 | | | 0.1625 | 0 | | | |
| 16 | 0.292682927 | | 0.595744681 | | | | 0.74251497 | | 0.368794326 | | | 0.0125 |  |  |  |  |
| 17 | 0.292682927 | | 0.595744681 | | | | 0.724550898 | | 0.290780142 | | | 0 |  |  |  |  |
| 18 | 0.292682927 | | 0.595744681 | | | | 0.724550898 | | 0.290780142 | | | 0 |  |  |  |  |
| 19 | 0.292682927 | | 0.329787234 | | | | 0.538922156 | | 6.38E-02 | | | 0 |  |  |  |  |
| 20 | 0.292682927 | | 0.244680851 | | | | 0.491017964 | | 2.84E-02 | | | 0 |  |  |  |  |
| 21 | 0.292682927 | | 0.244680851 | | | | 0.293413174 | | 7.09E-03 | | | 0 |  |  |  |  |
| 22 | 0.292682927 | | 3.19E-02 | | | | 0.185628743 | | 7.09E-03 | | | 0 |  |  |  |  |
| 23 | 0.292682927 | | 3.19E-02 | | | | 0.167664671 | | 7.09E-03 | | | 0 |  |  |  |  |
| 24 | 0.292682927 | | 0 | | | | 0.119760479 | | 7.09E-03 | | |  |  |  |  |  |
| 25 | 0.292682927 | | 0 | | | | 5.99E-03 | | 0 | | |  |  |  |  |  |
| 26 | 0.292682927 | | 0 | | | | 5.99E-03 | | 0 | | |  |  |  |  |  |
| 27 | 0.292682927 | | 0 | | | | 0 | | 0 | | |  |  |  |  |  |
| 28 | 0.207317073 | | 0 | | | | 0 | | 0 | | |  |  |  |  |  |
| 29 | 0.207317073 | | 0 | | | | 0 | | 0 | | |  |  |  |  |  |
| 30 | 0.207317073 | | 0 | | | | 0 | |  |  |  |  |  |  |  |  |
| 31 | 0.170731707 | | 0 | | | | 0 | |  |  |  |  |  |  |  |  |
| 32 | 6.10E-02 | | 0 | | | | 0 | |  |  |  |  |  |  |  |  |
| 33 | 0.024390244 | | 0 | | | | 0 | |  |  |  |  |  |  |  |  |
| 34 | 0.024390244 | | 0 | | | | 0 | |  |  |  |  |  |  |  |  |
| 35 | 0.024390244 | | 0 | | | | 0 | |  |  |  |  |  |  |  |  |
| 36 | 0.024390244 | | 0 | | | | 0 | |  |  |  |  |  |  |  |  |
| 37 | 1.22E-02 | |  | | | 0 | |  |  |  |  |  |  |  |  |  |
| 38 | 1.22E-02 | |  |  |  |  |  |  |  |  |  |  |  |  |  |  |
| 39 | 1.22E-02 | |  |  |  |  |  |  |  |  |  |  |  |  |  |  |
| 40 | 0 | |  |  |  |  |  |  |  |  |  |  |  |  |  |  |
| 41 | 0 | |  |  |  |  |  |  |  |  |  |  |  |  |  |  |
| 42 | 0 | |  |  |  |  |  |  |  |  |  |  |  |  |  |  |
| 43 | 0 | |  |  |  |  |  |  |  |  |  |  |  |  |  |  |
| 44 | 0 | |  |  |  |  |  |  |  |  |  |  |  |  |  |  |
| 45 | 0 | |  |  |  |  |  |  |  |  |  |  |  |  |  |  |
| 46 | 0 | |  |  |  |  |  |  |  |  |  |  |  |  |  |  |
| 47 | 0 | |  |  |  |  |  |  |  |  |  |  |  |  |  |  |
| 48 | 0 | |  |  |  |  |  |  |  |  |  |  |  |  |  |  |
| 49 | 0 | |  |  |  |  |  |  |  |  |  |  |  |  |  |  |

**Pupa**

| Temperature   \| Age  Survival rate \| \| --- \| \| | | 21℃ | 24℃ | | | | 27℃ | 30℃ | | | 33℃ | 36℃ | |  |
| --- | --- | --- | --- | --- | --- | --- | --- | --- | --- | --- | --- | --- | --- | --- | --- |
| 0 | 0 | | | 0 | | 0 | | | 0 | 0 | | 0 |  |  |
| 1 | 0 | | | 0 | | 0 | | | 0 | 0 | | 0 |  |  |
| 2 | 0 | | | 0 | | 0 | | | 0 | 0 | | 0 |  |  |
| 3 | 0 | | | 0 | | 0 | | | 0 | 0 | | 0 |  |  |
| 4 | 0 | | | 0 | | 0 | | | 0 | 0 | | 0 |  |  |
| 5 | 0 | | | 0 | | 0 | | | 0 | 0 | | 0 |  |  |
| 6 | 0 | | | 0 | | 0 | | | 0 | 0 | | 0.016 |  |  |
| 7 | 0 | | | 0 | | 0 | | | 0 | 0 | | 0.024 |  |  |
| 8 | 0 | | | 0 | | 0 | | | 0 | 0 | | 0.024 |  |  |
| 9 | 0 | | | 0 | | 0 | | | 0 | 0 | | 0.032 |  |  |
| 10 | 0 | | | 0 | | 0 | | | 0 | 0 | | 0.032 |  |  |
| 11 | 0 | | | 0 | | 0 | | | 0 | 0 | | 0.016 |  |  |
| 12 | 0 | | | 0 | | 0 | | | 0 | 0.025 | | 0.016 |  |  |
| 13 | 0 | | | 0 | | 0 | | | 3.55E-02 | 0.025 | | 0 |  |  |
| 14 | 0 | | | 0 | | 0 | | | 5.67E-02 | 0.1875 | | 0 |  |  |
| 15 | 0 | | | 0 | | 0 | | | 0.141843972 | 0.2 | | 0 |  |  |
| 16 | 0 | | | 0 | | 0 | | | 0.141843972 | 0.2875 | |  |  |  |
| 17 | 0 | | | 0 | | 1.80E-02 | | | 0.191489362 | 0.225 | |  |  |  |
| 18 | 0 | | | 0 | | 1.80E-02 | | | 0.141843972 | 0.15 | |  |  |  |
| 19 | 0 | | | 0.14893617 | | 0.19760479 | | | 0.113475177 | 0.0875 | |  |  |  |
| 20 | 0 | | | 0.191489362 | | 0.19760479 | | | 7.09E-02 | 0 | |  |  |  |
| 21 | 0 | | | 0.191489362 | | 0.329341317 | | | 6.38E-02 | 0 | |  |  |  |
| 22 | 0 | | | 0.35106383 | | 0.401197605 | | | 2.84E-02 | 0 | |  |  |  |
| 23 | 0 | | | 0.35106383 | | 0.407185629 | | | 1.42E-02 | 0 | |  |  |  |
| 24 | 0 | | | 0.382978723 | | 0.431137725 | | | 7.09E-03 |  |  |  |  |  |
| 25 | 0 | | | 0.276595745 | | 0.365269461 | | | 7.09E-03 |  |  |  |  |  |
| 26 | 0 | | | 0.191489362 | | 0.281437126 | | | 7.09E-03 |  |  |  |  |  |
| 27 | 0 | | | 6.38E-02 | | 0.167664671 | | | 7.09E-03 |  |  |  |  |  |
| 28 | 4.88E-02 | | | 2.13E-02 | | 6.59E-02 | | | 7.09E-03 |  |  |  |  |  |
| 29 | 4.88E-02 | | | 1.06E-02 | | 3.59E-02 | | | 0 |  |  |  |  |  |
| 30 | 4.88E-02 | | | 0 | | 2.40E-02 | | |  |  |  |  |  |  |
| 31 | 6.10E-02 | | | 0 | | 5.99E-03 | | |  |  |  |  |  |  |
| 32 | 8.54E-02 | | | 0 | | 0 | | |  |  |  |  |  |  |
| 33 | 7.32E-02 | | | 0 | | 0 | | |  |  |  |  |  |  |
| 34 | 6.10E-02 | | | 0 | | 0 | | |  |  |  |  |  |  |
| 35 | 4.88E-02 | | | 0 | | 0 | | |  |  |  |  |  |  |
| 36 | 4.88E-02 | | | 0 | | 0 | | |  |  |  |  |  |  |
| 37 | 3.66E-02 | | |  | 0 | | |  |  |  |  |  |  |  |
| 38 | 0.024390244 | | |  |  |  |  |  |  |  |  |  |  |  |
| 39 | 1.22E-02 | | |  |  |  |  |  |  |  |  |  |  |  |
| 40 | 0.024390244 | | |  |  |  |  |  |  |  |  |  |  |  |
| 41 | 0.024390244 | | |  |  |  |  |  |  |  |  |  |  |  |
| 42 | 0.024390244 | | |  |  |  |  |  |  |  |  |  |  |  |
| 43 | 1.22E-02 | | |  |  |  |  |  |  |  |  |  |  |  |
| 44 | 1.22E-02 | | |  |  |  |  |  |  |  |  |  |  |  |
| 45 | 0 | | |  |  |  |  |  |  |  |  |  |  |  |
| 46 | 0 | | |  |  |  |  |  |  |  |  |  |  |  |
| 47 | 0 | | |  |  |  |  |  |  |  |  |  |  |  |
| 48 | 0 | | |  |  |  |  |  |  |  |  |  |  |  |
| 49 | 0 | | |  |  |  |  |  |  |  |  |  |  |  |

**Female**

| Temperature   \| Age  Survival rate \| \| --- \| \| | | 21℃ | 24℃ | | | | 27℃ | 30℃ | | | 33℃ | 36℃ | | |  |
| --- | --- | --- | --- | --- | --- | --- | --- | --- | --- | --- | --- | --- | --- | --- | --- | --- |
| 0 | 0 | | | 0 | | 0 | | | 0 | 0 | | | 0 |  |  |
| 1 | 0 | | | 0 | | 0 | | | 0 | 0 | | | 0 |  |  |
| 2 | 0 | | | 0 | | 0 | | | 0 | 0 | | | 0 |  |  |
| 3 | 0 | | | 0 | | 0 | | | 0 | 0 | | | 0 |  |  |
| 4 | 0 | | | 0 | | 0 | | | 0 | 0 | | | 0 |  |  |
| 5 | 0 | | | 0 | | 0 | | | 0 | 0 | | | 0 |  |  |
| 6 | 0 | | | 0 | | 0 | | | 0 | 0 | | | 0 |  |  |
| 7 | 0 | | | 0 | | 0 | | | 0 | 0 | | | 0 |  |  |
| 8 | 0 | | | 0 | | 0 | | | 0 | 0 | | | 0 |  |  |
| 9 | 0 | | | 0 | | 0 | | | 0 | 0 | | | 0 |  |  |
| 10 | 0 | | | 0 | | 0 | | | 0 | 0 | | | 0 |  |  |
| 11 | 0 | | | 0 | | 0 | | | 0 | 0 | | | 0.008 |  |  |
| 12 | 0 | | | 0 | | 0 | | | 0 | 0 | | | 0.008 |  |  |
| 13 | 0 | | | 0 | | 0 | | | 0 | 0 | | | 0.016 |  |  |
| 14 | 0 | | | 0 | | 0 | | | 0 | 0 | | | 0.016 |  |  |
| 15 | 0 | | | 0 | | 0 | | | 0 | 0 | | | 0 |  |  |
| 16 | 0 | | | 0 | | 0 | | | 0 | 0.0125 | | |  |  |  |
| 17 | 0 | | | 0 | | 0 | | | 7.09E-03 | 0.0125 | | |  |  |  |
| 18 | 0 | | | 0 | | 0 | | | 2.13E-02 | 0.05 | | |  |  |  |
| 19 | 0 | | | 0 | | 0 | | | 0.04964539 | 0.05 | | |  |  |  |
| 20 | 0 | | | 0 | | 0 | | | 5.67E-02 | 0.075 | | |  |  |  |
| 21 | 0 | | | 0 | | 0 | | | 3.55E-02 | 0.05 | | |  |  |  |
| 22 | 0 | | | 0 | | 0 | | | 2.84E-02 | 0.025 | | |  |  |  |
| 23 | 0 | | | 0 | | 0 | | | 3.55E-02 | 0 | | |  |  |  |
| 24 | 0 | | | 0 | | 5.99E-03 | | | 3.55E-02 |  |  |  |  |  |  |
| 25 | 0 | | | 2.13E-02 | | 1.80E-02 | | | 2.84E-02 |  |  |  |  |  |  |
| 26 | 0 | | | 4.26E-02 | | 5.99E-02 | | | 2.13E-02 |  |  |  |  |  |  |
| 27 | 0 | | | 0.170212766 | | 0.107784431 | | | 2.13E-02 |  |  |  |  |  |  |
| 28 | 0 | | | 0.170212766 | | 0.119760479 | | | 7.09E-03 |  |  |  |  |  |  |
| 29 | 0 | | | 0.170212766 | | 0.137724551 | | | 0 |  |  |  |  |  |  |
| 30 | 0 | | | 0.138297872 | | 0.101796407 | | |  |  |  |  |  |  |  |
| 31 | 0 | | | 0.074468085 | | 7.78E-02 | | |  |  |  |  |  |  |  |
| 32 | 0 | | | 2.13E-02 | | 4.19E-02 | | |  |  |  |  |  |  |  |
| 33 | 0 | | | 1.06E-02 | | 3.59E-02 | | |  |  |  |  |  |  |  |
| 34 | 0 | | | 0 | | 0.02994012 | | |  |  |  |  |  |  |  |
| 35 | 0 | | | 0 | | 1.20E-02 | | |  |  |  |  |  |  |  |
| 36 | 0 | | | 0 | | 5.99E-03 | | |  |  |  |  |  |  |  |
| 37 | 0 | | |  | 0 | | |  |  |  |  |  |  |  |  |
| 38 | 0 | | |  |  |  |  |  |  |  |  |  |  |  |  |
| 39 | 1.22E-02 | | |  |  |  |  |  |  |  |  |  |  |  |  |
| 40 | 1.22E-02 | | |  |  |  |  |  |  |  |  |  |  |  |  |
| 41 | 1.22E-02 | | |  |  |  |  |  |  |  |  |  |  |  |  |
| 42 | 1.22E-02 | | |  |  |  |  |  |  |  |  |  |  |  |  |
| 43 | 0.024390244 | | |  |  |  |  |  |  |  |  |  |  |  |  |
| 44 | 1.22E-02 | | |  |  |  |  |  |  |  |  |  |  |  |  |
| 45 | 1.22E-02 | | |  |  |  |  |  |  |  |  |  |  |  |  |
| 46 | 1.22E-02 | | |  |  |  |  |  |  |  |  |  |  |  |  |
| 47 | 0 | | |  |  |  |  |  |  |  |  |  |  |  |  |
| 48 | 0 | | |  |  |  |  |  |  |  |  |  |  |  |  |
| 49 | 0 | | |  |  |  |  |  |  |  |  |  |  |  |  |

**Male**

| Temperature   \| Age  Survival rate \| \| --- \| \| | | 21℃ | 24℃ | | | | 27℃ | | | 30℃ | | 33℃ | 36℃ | |
| --- | --- | --- | --- | --- | --- | --- | --- | --- | --- | --- | --- | --- | --- | --- | --- |
| 0 | 0 | | | 0 | | 0 | | | 0 | | 0 | | 0 |  |
| 1 | 0 | | | 0 | | 0 | | | 0 | | 0 | | 0 |  |
| 2 | 0 | | | 0 | | 0 | | | 0 | | 0 | | 0 |  |
| 3 | 0 | | | 0 | | 0 | | | 0 | | 0 | | 0 |  |
| 4 | 0 | | | 0 | | 0 | | | 0 | | 0 | | 0 |  |
| 5 | 0 | | | 0 | | 0 | | | 0 | | 0 | | 0 |  |
| 6 | 0 | | | 0 | | 0 | | | 0 | | 0 | | 0 |  |
| 7 | 0 | | | 0 | | 0 | | | 0 | | 0 | | 0 |  |
| 8 | 0 | | | 0 | | 0 | | | 0 | | 0 | | 0 |  |
| 9 | 0 | | | 0 | | 0 | | | 0 | | 0 | | 0 |  |
| 10 | 0 | | | 0 | | 0 | | | 0 | | 0 | | 0 |  |
| 11 | 0 | | | 0 | | 0 | | | 0 | | 0 | | 0.008 |  |
| 12 | 0 | | | 0 | | 0 | | | 0 | | 0 | | 0.008 |  |
| 13 | 0 | | | 0 | | 0 | | | 0 | | 0 | | 0.008 |  |
| 14 | 0 | | | 0 | | 0 | | | 0 | | 0 | | 0.008 |  |
| 15 | 0 | | | 0 | | 0 | | | 0 | | 0 | | 0 |  |
| 16 | 0 | | | 0 | | 0 | | | 0 | | 0.0125 | |  |  |
| 17 | 0 | | | 0 | | 0 | | | 1.42E-02 | | 0.0625 | |  |  |
| 18 | 0 | | | 0 | | 0 | | | 0.04964539 | | 0.1 | |  |  |
| 19 | 0 | | | 0 | | 0 | | | 7.80E-02 | | 0.125 | |  |  |
| 20 | 0 | | | 0 | | 0 | | | 9.22E-02 | | 0.1125 | |  |  |
| 21 | 0 | | | 0 | | 0 | | | 7.80E-02 | | 0.0625 | |  |  |
| 22 | 0 | | | 0 | | 0 | | | 6.38E-02 | | 0.0375 | |  |  |
| 23 | 0 | | | 0 | | 1.20E-02 | | | 0.04964539 | | 0 | |  |  |
| 24 | 0 | | | 0 | | 2.40E-02 | | | 2.84E-02 | |  |  |  |  |
| 25 | 0 | | | 0.074468085 | | 4.79E-02 | | | 1.42E-02 | |  |  |  |  |
| 26 | 0 | | | 0.138297872 | | 5.99E-02 | | | 0 | |  |  |  |  |
| 27 | 0 | | | 0.138297872 | | 0.125748503 | | | 0 | |  |  |  |  |
| 28 | 0 | | | 0.159574468 | | 0.119760479 | | | 0 | |  |  |  |  |
| 29 | 0 | | | 0.159574468 | | 0.113772455 | | | 0 | |  |  |  |  |
| 30 | 0 | | | 0.138297872 | | 0.101796407 | | |  |  |  |  |  |  |
| 31 | 0 | | | 0.127659574 | | 4.79E-02 | | |  |  |  |  |  |  |
| 32 | 1.22E-02 | | | 0.106382979 | | 0.02994012 | | |  |  |  |  |  |  |
| 33 | 1.22E-02 | | | 6.38E-02 | | 2.40E-02 | | |  |  |  |  |  |  |
| 34 | 1.22E-02 | | | 3.19E-02 | | 1.20E-02 | | |  |  |  |  |  |  |
| 35 | 0.024390244 | | | 1.06E-02 | | 5.99E-03 | | |  |  |  |  |  |  |
| 36 | 0.024390244 | | | 0 | | 0 | | |  |  |  |  |  |  |
| 37 | 3.66E-02 | | |  | 0 | | |  |  |  |  |  |  |  |
| 38 | 4.88E-02 | | |  |  |  |  |  |  |  |  |  |  |  |
| 39 | 4.88E-02 | | |  |  |  |  |  |  |  |  |  |  |  |
| 40 | 4.88E-02 | | |  |  |  |  |  |  |  |  |  |  |  |
| 41 | 0.024390244 | | |  |  |  |  |  |  |  |  |  |  |  |
| 42 | 1.22E-02 | | |  |  |  |  |  |  |  |  |  |  |  |
| 43 | 1.22E-02 | | |  |  |  |  |  |  |  |  |  |  |  |
| 44 | 1.22E-02 | | |  |  |  |  |  |  |  |  |  |  |  |
| 45 | 1.22E-02 | | |  |  |  |  |  |  |  |  |  |  |  |
| 46 | 1.22E-02 | | |  |  |  |  |  |  |  |  |  |  |  |
| 47 | 1.22E-02 | | |  |  |  |  |  |  |  |  |  |  |  |
| 48 | 1.22E-02 | | |  |  |  |  |  |  |  |  |  |  |  |
| 49 | 0 | | |  |  |  |  |  |  |  |  |  |  |  |
