# Supplementary material for: Age-stage, two-sex life table of Parapoynx crisonalis (Lepidoptera: Pyralidae) at different temperatures
Source: PLoS One. 2017 Mar 6;12(3):e0173380. doi: 10.1371/journal.pone.0173380 (PMC5338836; doi:10.1371/journal.pone.0173380)
Supplement: S3 Data Set — (DOCX) [file pone.0173380.s003.docx]

**S3 Data Set. Fig. 3 Age-specific survival rate, age-stage-specific fecundity and age-specific fecundity of *Parapoynx crisonalis*.**

**21℃**

| Age   \| Parameter  Value \| \| --- \| \| | *l_x_* | *f_x_* | *m_x_* |
| --- | --- | --- | --- | --- |
| 0 | 1 | 0 | 0 |
| 1 | 1 | 0 | 0 |
| 2 | 1 | 0 | 0 |
| 3 | 1 | 0 | 0 |
| 4 | 1 | 0 | 0 |
| 5 | 1 | 0 | 0 |
| 6 | 1 | 0 | 0 |
| 7 | 0.317073171 | 0 | 0 |
| 8 | 0.317073171 | 0 | 0 |
| 9 | 0.292682927 | 0 | 0 |
| 10 | 0.292682927 | 0 | 0 |
| 11 | 0.292682927 | 0 | 0 |
| 12 | 0.292682927 | 0 | 0 |
| 13 | 0.292682927 | 0 | 0 |
| 14 | 0.292682927 | 0 | 0 |
| 15 | 0.292682927 | 0 | 0 |
| 16 | 0.292682927 | 0 | 0 |
| 17 | 0.292682927 | 0 | 0 |
| 18 | 0.292682927 | 0 | 0 |
| 19 | 0.292682927 | 0 | 0 |
| 20 | 0.292682927 | 0 | 0 |
| 21 | 0.292682927 | 0 | 0 |
| 22 | 0.292682927 | 0 | 0 |
| 23 | 0.292682927 | 0 | 0 |
| 24 | 0.292682927 | 0 | 0 |
| 25 | 0.292682927 | 0 | 0 |
| 26 | 0.292682927 | 0 | 0 |
| 27 | 0.292682927 | 0 | 0 |
| 28 | 0.256097561 | 0 | 0 |
| 29 | 0.256097561 | 0 | 0 |
| 30 | 0.256097561 | 0 | 0 |
| 31 | 0.231707317 | 0 | 0 |
| 32 | 0.158536585 | 0 | 0 |
| 33 | 0.109756098 | 0 | 0 |
| 34 | 9.76E-02 | 0 | 0 |
| 35 | 9.76E-02 | 0 | 0 |
| 36 | 9.76E-02 | 0 | 0 |
| 37 | 8.54E-02 | 0 | 0 |
| 38 | 8.54E-02 | 0 | 0 |
| 39 | 8.54E-02 | 0 | 0 |
| 40 | 8.54E-02 | 8 | 1.142857143 |
| 41 | 6.10E-02 | 0 | 0 |
| 42 | 4.88E-02 | 0 | 0 |
| 43 | 4.88E-02 | 0 | 0 |
| 44 | 3.66E-02 | 6 | 2 |
| 45 | 0.024390244 | 0 | 0 |
| 46 | 0.024390244 | 0 | 0 |
| 47 | 1.22E-02 | 0 | 0 |
| 48 | 1.22E-02 | 0 | 0 |
| 49 | 0 | 0 | 0 |

**24℃**

| Age   \| Parameter  Value \| \| --- \| \| | *l_x_* | *f_x_* | *m_x_* |
| --- | --- | --- | --- | --- |
| 0 | 1 | 0 | 0 |
| 1 | 1 | 0 | 0 |
| 2 | 1 | 0 | 0 |
| 3 | 1 | 0 | 0 |
| 4 | 1 | 0 | 0 |
| 5 | 1 | 0 | 0 |
| 6 | 0.595744681 | 0 | 0 |
| 7 | 0.595744681 | 0 | 0 |
| 8 | 0.595744681 | 0 | 0 |
| 9 | 0.595744681 | 0 | 0 |
| 10 | 0.595744681 | 0 | 0 |
| 11 | 0.595744681 | 0 | 0 |
| 12 | 0.595744681 | 0 | 0 |
| 13 | 0.595744681 | 0 | 0 |
| 14 | 0.595744681 | 0 | 0 |
| 15 | 0.595744681 | 0 | 0 |
| 16 | 0.595744681 | 0 | 0 |
| 17 | 0.595744681 | 0 | 0 |
| 18 | 0.595744681 | 0 | 0 |
| 19 | 0.478723404 | 0 | 0 |
| 20 | 0.436170213 | 0 | 0 |
| 21 | 0.436170213 | 0 | 0 |
| 22 | 0.382978723 | 0 | 0 |
| 23 | 0.382978723 | 0 | 0 |
| 24 | 0.382978723 | 0 | 0 |
| 25 | 0.372340426 | 0 | 0 |
| 26 | 0.372340426 | 0 | 0 |
| 27 | 0.372340426 | 25.4375 | 11.62857143 |
| 28 | 0.35106383 | 31.5625 | 15.3030303 |
| 29 | 0.340425532 | 227.375 | 113.6875 |
| 30 | 0.276595745 | 69.38461538 | 34.69230769 |
| 31 | 0.20212766 | 96 | 35.36842105 |
| 32 | 0.127659574 | 57.5 | 9.583333333 |
| 33 | 0.074468085 | 48 | 6.857142857 |
| 34 | 3.19E-02 | 0 | 0 |
| 35 | 1.06E-02 | 0 | 0 |
| 36 | 0 | 0 | 0 |

**27℃**

| Age   \| Parameter  Value \| \| --- \| \| | *l_x_* | *f_x_* | *m_x_* |
| --- | --- | --- | --- | --- |
| 0 | 1 | 0 | 0 |
| 1 | 1 | 0 | 0 |
| 2 | 1 | 0 | 0 |
| 3 | 1 | 0 | 0 |
| 4 | 0.74251497 | 0 | 0 |
| 5 | 0.74251497 | 0 | 0 |
| 6 | 0.74251497 | 0 | 0 |
| 7 | 0.74251497 | 0 | 0 |
| 8 | 0.74251497 | 0 | 0 |
| 9 | 0.74251497 | 0 | 0 |
| 10 | 0.74251497 | 0 | 0 |
| 11 | 0.74251497 | 0 | 0 |
| 12 | 0.74251497 | 0 | 0 |
| 13 | 0.74251497 | 0 | 0 |
| 14 | 0.74251497 | 0 | 0 |
| 15 | 0.74251497 | 0 | 0 |
| 16 | 0.74251497 | 0 | 0 |
| 17 | 0.74251497 | 0 | 0 |
| 18 | 0.74251497 | 0 | 0 |
| 19 | 0.736526946 | 0 | 0 |
| 20 | 0.688622754 | 0 | 0 |
| 21 | 0.622754491 | 0 | 0 |
| 22 | 0.586826347 | 0 | 0 |
| 23 | 0.586826347 | 0 | 0 |
| 24 | 0.580838323 | 0 | 0 |
| 25 | 0.437125749 | 0 | 0 |
| 26 | 0.407185629 | 34.2 | 5.029411765 |
| 27 | 0.401197605 | 12.66666667 | 3.402985075 |
| 28 | 0.305389222 | 72.15 | 28.29411765 |
| 29 | 0.28742515 | 88.39130435 | 42.35416667 |
| 30 | 0.22754491 | 74.41176471 | 33.28947368 |
| 31 | 0.131736527 | 54.84615385 | 32.40909091 |
| 32 | 7.19E-02 | 122.4285714 | 71.41666667 |
| 33 | 5.99E-02 | 144.5 | 86.7 |
| 34 | 4.19E-02 | 121 | 86.42857143 |
| 35 | 1.80E-02 | 69.5 | 46.33333333 |
| 36 | 5.99E-03 | 29 | 29 |
| 37 | 0 | 0 | 0 |

**30℃**

| Age   \| Parameter  Value \| \| --- \| \| | *l_x_* | *f_x_* | *m_x_* |
| --- | --- | --- | --- | --- |
| 0 | 1 | 0 | 0 |
| 1 | 1 | 0 | 0 |
| 2 | 1 | 0 | 0 |
| 3 | 1 | 0 | 0 |
| 4 | 0.666666667 | 0 | 0 |
| 5 | 0.666666667 | 0 | 0 |
| 6 | 0.666666667 | 0 | 0 |
| 7 | 0.666666667 | 0 | 0 |
| 8 | 0.666666667 | 0 | 0 |
| 9 | 0.666666667 | 0 | 0 |
| 10 | 0.666666667 | 0 | 0 |
| 11 | 0.666666667 | 0 | 0 |
| 12 | 0.666666667 | 0 | 0 |
| 13 | 0.609929078 | 0 | 0 |
| 14 | 0.609929078 | 0 | 0 |
| 15 | 0.510638298 | 0 | 0 |
| 16 | 0.510638298 | 0 | 0 |
| 17 | 0.503546099 | 0 | 0 |
| 18 | 0.503546099 | 0 | 0 |
| 19 | 0.304964539 | 37 | 6.023255814 |
| 20 | 0.24822695 | 3 | 0.685714286 |
| 21 | 0.184397163 | 11.6 | 2.230769231 |
| 22 | 0.127659574 | 86 | 19.11111111 |
| 23 | 0.106382979 | 80.4 | 26.8 |
| 24 | 7.80E-02 | 33.4 | 15.18181818 |
| 25 | 0.04964539 | 87.5 | 50 |
| 26 | 2.84E-02 | 57.66666667 | 43.25 |
| 27 | 2.84E-02 | 166.3333333 | 124.75 |
| 28 | 1.42E-02 | 32 | 16 |
| 29 | 0 | 0 | 0 |

**33℃**

| Age   \| Parameter  Value \| \| --- \| \| | *l_x_* | *f_x_* | *m_x_* |
| --- | --- | --- | --- | --- |
| 0 | 1 | 0 | 0 |
| 1 | 1 | 0 | 0 |
| 2 | 1 | 0 | 0 |
| 3 | 0.4875 | 0 | 0 |
| 4 | 0.4875 | 0 | 0 |
| 5 | 0.4875 | 0 | 0 |
| 6 | 0.4875 | 0 | 0 |
| 7 | 0.4875 | 0 | 0 |
| 8 | 0.4875 | 0 | 0 |
| 9 | 0.4875 | 0 | 0 |
| 10 | 0.4875 | 0 | 0 |
| 11 | 0.4875 | 0 | 0 |
| 12 | 0.4875 | 0 | 0 |
| 13 | 0.4875 | 0 | 0 |
| 14 | 0.3875 | 0 | 0 |
| 15 | 0.3625 | 0 | 0 |
| 16 | 0.325 | 0 | 0 |
| 17 | 0.3 | 0 | 0 |
| 18 | 0.3 | 2.5 | 0.416666667 |
| 19 | 0.2625 | 0 | 0 |
| 20 | 0.1875 | 1.833333333 | 0.733333333 |
| 21 | 0.1125 | 0 | 0 |
| 22 | 0.0625 | 0 | 0 |
| 23 | 0 | 0 | 0 |

**36℃**

| Age   \| Parameter  Value \| \| --- \| \| | *l_x_* | *f_x_* | *m_x_* |
| --- | --- | --- | --- | --- |
| 0 | 1 | 0 | 0 |
| 1 | 1 | 0 | 0 |
| 2 | 0.592 | 0 | 0 |
| 3 | 0.592 | 0 | 0 |
| 4 | 0.592 | 0 | 0 |
| 5 | 0.592 | 0 | 0 |
| 6 | 0.032 | 0 | 0 |
| 7 | 0.032 | 0 | 0 |
| 8 | 0.032 | 0 | 0 |
| 9 | 0.032 | 0 | 0 |
| 10 | 0.032 | 0 | 0 |
| 11 | 0.032 | 0 | 0 |
| 12 | 0.032 | 0 | 0 |
| 13 | 0.024 | 3 | 2 |
| 14 | 0.024 | 4 | 2.666666667 |
| 15 | 0 | 0 | 0 |
